# Teaching Oral Hygiene to Recruits

**Published:** 1918-03

**Authors:** H. J. Brachman

**Affiliations:** U. of P., 1909, MT. Clemens, Mich.


### TEACHING ORAL HYGIENE TO RECRUITS

BY H. J. BRACHMAN, D.D.S. U. OF P., 1909, MT. CLEMENS, MICH.

Dr. Brachman has learned one of the most important of all arts, that of talking to people so that they can understand. It would be easy for most of us if we didn't think that we ought to do something extraordinary when we talk to people. If we had the courage just to dare to be ourselves and to say the natural things, we should help people much more than we do.

Dr. Brachman's suggestion that dentists who have conscientious scruples against subscribing to the tobacco fund, might establish a "toothbrush and toothpaste fund" with the same recipients, is excellent.—*Editor Dental Digest.*

With this country at war and a large number of dental surgeons in active service "with the colors," the experiences of a colleague may be of interest to members of the dental profession, particularly the suggestions intended to produce. the cooperation of the personnel of our forces with the dental surgeons and incidentally produce a mutual understanding that should prove of mutual benefit.

I arrived at my post, "somewhere in America" one Monday morning, just three weeks ago, reported to the commanding officer and then to the chief surgeon for duty. He introduced me to my associates at the post hospital, two other medical officers, and they all acted very kindly and cooperated with me in every manner. A room in the building was assigned to me for a dental office, the number of men here having been augmented only a few days before to approximately the quota required by army regulations to necessitate the services of a dental surgeon.

After having been comfortably quartered, I was anxious to begin my work, and, at the suggestion of the chief surgeon, wired my requisition for a portable outfit, mailing the regulation order the same day.

Realizing that there would be a necessary delay, due to the great number of demands for outfits of similar nature that had preceded mine, I decided to spend the time that would elapse by examining the mouths of the men in squads of twelve, after giving them a sort of heart-to-heart talk on the care of the teeth and the reason thereof. Accordingly, I secured some examination charts, mouth mirrors and explorers and announced that I would be ready to proceed as per the above, the following Monday morning.

A squad of twelve men reported at my office each hour and from the first they paid strict attention to what I had to say, and starting with an eight-minute talk, I found before the week was over that I was giving a thirty-minute one that seemed to impress the "boys," as their interesting questions at the end of each session led me to believe. I found that I could not examine the mouths of all the men in the entire outfit before the arrival of my instruments, and being anxious to give them all a few words of advice on the care of the teeth, I arranged and talked to the balance in squads of 100 at 5:30 each evening at their respective mess halls, immediately after supper, so that my talk would not interfere with any military or social duties that may have been scheduled.

To make a long story short, a great many of the men began to take more interest in the care of their mouths and teeth. The reports of the top sergeants informed me of increased activity in the tooth-brushing industry, and I personally determined from the post exchange and the druggists in the town proper that the sale of tooth-brushes, paste, powder and mouth wash had taken a big boost. I felt that my time had been well spent and that the results more than justified my humble efforts.

In my talk I would start by informing the men that, being in the army, they naturally were supposed to be able to stand pain, exposure, etc. When a slight pain in a tooth occurred, as army men, they would not pay any attention to it until too late and then have it extracted, having somewhat considered the care of the teeth as savoring of vanity, and not absolutely necessary, as there was no specific time scheduled for it in army routine. I would then inform them of how necessary bridge work, to replace lost teeth, would impair the longevity of the abutments. The crowning of a tooth with gold covering a multitude of sins when it could be filled was likened unto the ostrich who, because he stuck his head in sand, and could not see any one, imagined that no one could see him.

For the sake of comparison, the abscesses, or gum-boils, as they called them, were likened unto the abscess on a bone felon that they would suck. Rather a grewsome comparison, but nevertheless a true one, and decayed teeth and their effect on the normal teeth was explained by the effect of the "spoiled apple in a barrel of good apples."

The mouth with its teeth was spoken of as the carburetor of the body, for in the same manner that the carburetor of a motor would break up the gasoline so that the necessary power would be forthcoming from the cylinders to propel the vehicle, the teeth prepare the food to be digested by the digestive tract to produce the necessary strength and stamina to withstand the rigors of army life, together with its exposure to inclement weather. Therefore the importance of keeping the mouth in normal condition was made obvious. The fact that a well-nourished body increases the immunity against infection and insures a more speedy recovery in the event of being wounded was brought to their attention with forcible illustrations.

In order to savor of a military routine, the brushing of the teeth was called, "the toothbrush setting-up exercise" for the reason that in the same manner that the setting-up exercise creates a healthy circulation of the blood, the proper toothbrush manipulation creates a healthy circulation in the gum tissues with just as exhilarating effects. There were thirteen figures (hard luck to deposits on the teeth), and they were requested to go through this exercise at least twice each day, night and morning. They were reminded that the germ is possibly more deadly than the German, and the reason for brushing their teeth before retiring was to allow the body to charge up on energy properly while "at ease" in the same manner that any battery will be charged up when connected with a charging station.

Quite a number of men who have seen service "over there" inform me that the natural tendency is to neglect the teeth, and this is a condition that we must try to guard against among men.

If I may be so bold, I would suggest that the dental surgeons in active service take the men into their confidence with heart-to-heart talks as outlined.

As there are a great many people who would like to contribute to a personal comfort movement for the men in service, but as they do not approve of the use of tobacco, they have not subscribed to that fund, and I would like to suggest that the civilian members of the dental profession inaugurate a movement in their respective communities for a "toothbrush and toothpaste fund," worked along similar lines, in order to bring the absolute necessity of the care of the teeth to the minds of the men in a forcible manner, a pamphlet embodying a heart-to-heart talk on this subject could be enclosed in each package.

In order to avoid misunderstanding, I do not mean to infer by the above that the men are neglecting their teeth, but I do want to state that a movement such as this would properly educate them for the future so that their mouths would not be in the poor condition that inevitably results from a sojourn in the trenches where proper dental care, if not firmly drilled into the minds of the men, is so likely to be neglected, as the experiences of our allied dental officers teach us.—*Dental Digest* for January, 1918.

